# Postoperative brain volumes are associated with one-year neurodevelopmental outcome in children with severe congenital heart disease

**DOI:** 10.1038/s41598-019-47328-9

**Published:** 2019-07-26

**Authors:** Eliane Meuwly, Maria Feldmann, Walter Knirsch, Michael von Rhein, Kelly Payette, Hitendu Dave, Ruth O’ Gorman Tuura, Raimund Kottke, Cornelia Hagmann, Beatrice Latal, András Jakab, on behalf of the Research Group Heart and Brain*, Rabia Liamlahi, Annette Hackenberg, Oliver Kretschmar, Christian Kellenberger, Christoph Bürki, Markus Weiss

**Affiliations:** 1 Child Development Centre, University Children’s Hospital, Zurich, Switzerland; 2 Children’s Research Centre, University Children’s Hospital Zurich, Zürich, Switzerland; 3 Paediatric Cardiology, Paediatric Heart Centre, Department of Surgery, University Children’s Hospital Zurich, Zürich, Switzerland; 4 Centre for MR Research, University Children’s Hospital of Zurich, Zürich, Switzerland; 5 Paediatric Cardiovascular Surgery, Paediatric Heart Centre, Department of Surgery, University Children’s Hospital Zurich, Zürich, Switzerland; 6 Department of Diagnostic Imaging, University Children’s Hospital Zurich, Zürich, Switzerland; 7 Department of Neonatology and Paediatric Intensive care, University Children’s Hospital Zurich, Zürich, Switzerland; 8 Department of Pediatric Neurology, University Children’s Hospital, Zürich, Switzerland; 9 Department of Anesthesiology, University Children’s Hospital Zurich, Zurich, Switzerland

**Keywords:** Outcomes research, Paediatric research

## Abstract

Children with congenital heart disease (CHD) remain at risk for neurodevelopmental impairment despite improved perioperative care. Our prospective cohort study aimed to determine the relationship between perioperative brain volumes and neurodevelopmental outcome in neonates with severe CHD. Pre- and postoperative cerebral MRI was acquired in term born neonates with CHD undergoing neonatal cardiopulmonary bypass surgery. Brain volumes were measured using an atlas prior-based automated method. One-year neurodevelopmental outcome was assessed with the Bayley-III. CHD infants (n = 77) had lower pre- and postoperative total and regional brain volumes compared to controls (n = 44, all p < 0.01). CHD infants had poorer cognitive and motor outcome (p ≤ 0.0001) and a trend towards lower language composite score compared to controls (p = 0.06). Larger total and selected regional postoperative brain volumes were found to be associated with better cognitive and language outcomes (all p < 0.04) at one year. This association was independent of length of intensive care unit stay for total, cortical, temporal, frontal and cerebellar volumes. Therefore, reduced cerebral volume in CHD neonates undergoing bypass surgery may serve as a biomarker for impaired outcome.

## Introduction

Congenital heart disease (CHD) is the most common congenital malformation in childhood^1^. Despite dramatic improvements in peri- and intraoperative care that have led to an increase in survival rates, children with severe CHD remain at risk for neurodevelopmental impairment^1,2^. Cognitive, language and motor functions may be impaired during early childhood^3–5^, but impairments often persist and include deficits in higher order cognitive functions in adolescence^6,7^.

Cerebral magnetic resonance imaging (MRI) studies in neonates with CHD have shown a high incidence of punctate pre-^2,8,9^ and postoperative white matter injuries (WMI)^2,10^ and strokes^2,11,12^. Furthermore, delayed brain maturation and impaired fetal brain growth has been described in this population^13–15^, indicative of widespread alterations in brain maturation and an increased vulnerability to brain injury.

Volumetric analyses of neonatal cerebral MRI examinations have revealed that infants with severe CHD have smaller brain volumes compared to healthy infants^3,8^, with all brain regions being equally affected^3^.

There is mounting evidence that structural brain abnormalities in neonates with CHD are associated with poorer neurodevelopmental outcome^4,11,12,16,17^. However, while total brain volume reduction has been shown to correlate with neurobehaviour prior to neonatal surgery^4^, the impact of neonatal brain volume reduction on neurodevelopmental outcome in early childhood is yet unknown. Elucidating the association between total and regional brain volume reduction and specific neurodevelopmental outcomes may enable us to determine the antecedents of later altered development^3^.

The aim of our study was to examine neonatal brain volumes in CHD subjects compared to healthy controls and to assess the association of pre- and postoperative total and regional brain volumes with one-year neurodevelopmental outcome in children with severe CHD. Our primary hypothesis is that lower total and regional brain volumes are associated with poorer neurodevelopmental outcome, independent of patient-specific and perioperative risk factors.

## Methods

### Population

This study is part of an ongoing prospective cohort study investigating neurodevelopmental outcome and neuroimaging findings in infants operated for CHD^3^. Between December 2009 and August 2016, term born neonates (>36 weeks of gestation) with severe CHD undergoing cardiopulmonary bypass surgery (CPB) were consecutively enrolled in the study. Written informed consent was provided from the parents or legal guardians and the study was approved by the ethical committee of Kanton Zürich, Switzerland (Kantonale Ethikkommission Zürich). The study was carried out in accordance with the principles enunciated in the Declaration of Helsinki and the guidelines of Good Clinical Practice. Neonates were enrolled in the study after being admitted to the paediatric cardiac intensive care unit (ICU). Neonates with a suspected or confirmed genetic disorder or syndrome were excluded. Infants included in the study underwent cerebral MRI before and after surgery and a neurodevelopmental assessment at 12 months of age, using the Bayley Scales of Infant and Toddler Development, Third Edition (Bayley-III). The Bayley–III assesses different developmental domains and provides three main composite scores: the cognitive composite score (CCS), the language composite score (LCS) and the motor composite score (MCS) with a mean score of 100 and a standard deviation of + /− 15. The Bayley-III was administered by trained developmental paediatricians. As the neurodevelopmental follow up of children with congenital heart disease is performed routinely as part of a clinical follow up program, developmental paediatricians were not blinded to clinical and MRI data.

Socioeconomic status (SES) was estimated based on maternal education and paternal occupation^18^, yielding a score ranging from 2 to 12 with higher scores indicating higher SES.

Healthy term born infants were recruited between 2011 and 2016 as controls from the well-baby maternity unit at the University Hospital Zurich. None of the control infants had any cerebral lesions on MRI, and none of the controls had been admitted to the neonatal unit.

### Cerebral MRI and image post processing

Neonatal cerebral MRI was performed during natural sleep on a 3.0 T clinical MRI scanner using an 8-channel head coil (GE Signa MR750). Ear plugs (attenuation: 24 dB; Earsoft; Aearo) and Minimuffs (attenuation: 7 dB; Natus) were applied for noise protection. Oxygen saturation was monitored during scanning, and a neonatologist and a neonatal nurse were present during each MRI investigation. Structural MRI sequences included 2D fast spin-echo (FSE) T2-weighted sequences acquired in axial, sagittal and coronal planes. The sequence parameters for the anatomical MRI were the following. TE/TR: 97/5900 ms, flip angle: 90°, number of averages: 2, acquisition matrix: 512 * 320, in-plane voxel dimensions: 0.7 * 0.7 mm resampled to 0.35 * 0.35 mm, slice thickness: 2.5 mm, slice gap: 0.2 mm. A slice gap was used to reduce the effects of slice cross-talk when scanning with the 2D FSE sequence, and the effective slice thickness after image conversion and reconstruction was 2.7 mm. Anatomical MRI was followed by diffusion tensor imaging, proton MR spectroscopy and arterial spin labelling sequences. Further details about the imaging setting and MRI sequence parameters for this ongoing study have been reported previously^10^.

Each MRI scan was scored for the presence of brain abnormalities by a trained neuroradiologist blinded to all clinical characteristics^10^. During the course of the study, a scanner software update was performed. Therefore, the cohort was divided into two groups (MRI Cohort 1 and 2) according to the scanner upgrade status, and this variable was included in the statistical tests as a covariate.

**Figure 1:**
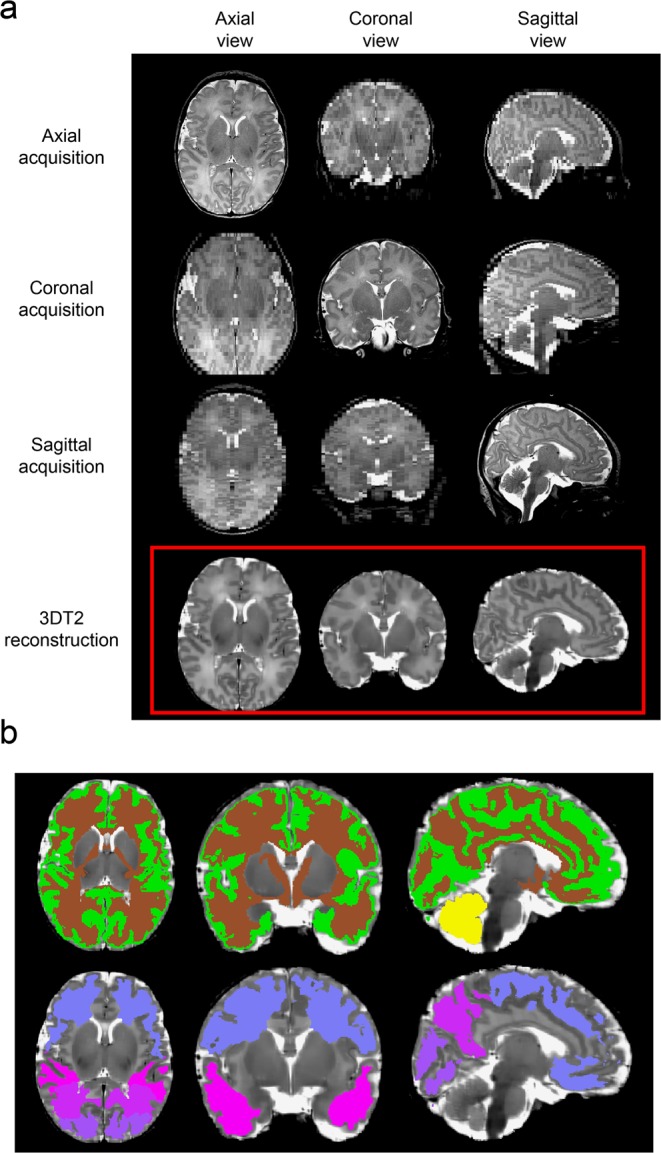
3D reconstruction and brain segmentation. (**a**) Reconstruction of 3D T2-weighted images (bottom row) from three orthogonally acquired images, (**b**) example segmentations: cortex, white matter and cerebellum (top row), and four-lobe segmentation (bottom row).

An automatic image analysis workflow was utilized to measure total and regional brain volumes in each neonate. First, T2-weighted anatomical images were re-sampled to a super-resolution image using the principles described in previous reports^19^. Non-brain tissue parts of the axial, coronal and sagittal T2-weighted images were removed by applying image masks generated from an age-specific neonatal neuroanatomical atlas. The masked, axial and coronal images were co-registered to the sagittal image using a mutual information based affine and non-linear registration as implemented in the Niftireg image registration package^20^. A slice-wise reconstruction step, which is usually employed during super-resolution reconstructions^21^, was skipped due to the relative scarcity of subject motion, and since any motion-corrupted scans had been repeated. Bias field correction of the images was performed by the N4ITK filter in the Slicer 3D software^22^, while further steps of the re-sampling were carried out using the BTK Toolkit^23^. The original, three-plane T2 images and the 3DT2 reconstruction are illustrated in Fig. 1a.

Anatomical priors from the gestational age-specific neonatal anatomical atlas ALBERTs^24^ were matched to the subjects reconstructed images using a freeform non-linear deformation (reg_f3d command in the Niftireg software) with a fine transformation grid of 8 × 8 × 8 mm. A penalty term was applied with a bending energy setting of 0.0035 to allow for more accurate alignment between the template and subject’s images. The anatomical masks of white matter, grey matter (cortex only) and lobar subdivisions of the supratentorial white matter (frontal, parietal, temporal and occipital lobe) and cerebellum were matched to the T2-weighted super-resolution images. Total brain (parenchymal) volume was estimated by constructing a whole-brain mask, which included the diencephalon, cortical mantle, and supratentorial white matter, but not the intra- and extra axial cerebrospinal fluid spaces or cerebellum. The patient-specific priors^25^ were thresholded at 50% probability, and their volume was stored for the statistical evaluations (example segmentations: Fig. 1b).

To validate the automated atlas-based volumetry workflow against manual volumetry, a stereological analysis of a sub-set of the study sample was performed. Stereology was carried out using the EasyMeasure software package^26^, following a method that has previously been described in detail elsewhere^27^. The validation showed good agreement between the two methods, with a Pearson correlation coefficient ranging from 0.930 (p < 0.01) for the total brain volume and 0.827 (p < 0.01) for the cerebellar volume. In-depth details and results of the validation process are given in the Supplementary Information.

Following this initial validation, all subsequent analyses including brain volumes were performed by the automated pipeline.

### Statistical analyses

All analyses were conducted using the statistical software R, Version 3.5.0^28^. Subject demographics are listed in Table 1 as mean and standard deviation (SD) for normally distributed continuous variables and median and interquartile range (IQR) for non-normally distributed continuous variables. Categorical variables are reported as proportions. Groupwise comparisons for continuous variables were performed by t tests and Man-Whitney U tests as appropriate for the sample distributions, and comparisons for categorical variables were assessed using Fisher’s exact test. To quantify the associations between brain volumes and neurodevelopmental outcome, we calculated a series of multiple linear regression models adjusted for postmenstrual age at MRI, SES, sex and MRI cohort, with the latter accounting for a software update on the scanner during the time of the study. To further correct for possible confounds associated with the length of ICU stay, this variable was consecutively introduced into the model. Square root transformation for dependent variables such as brain volumes and Bayley-III composite scores was performed to meet the assumptions of multiple linear regression models. Pearson correlation coefficients were calculated to determine the relationship between potential risk factors and total brain volumes in infants with CHD.

**Table 1:** Characteristics of infants with CHD and healthy controls. M, Median; m, mean; IQR, interquartile range; SD, standard deviation; CHD, congenital heart disease; GA, gestational age; ICU, intensive care unit; SES, socioeconomic status; MRI, magnetic resonance imaging; NA, not applicable. Groupwise comparison for categorical variables was performed by Fisher’s exact test and for continuous variables t test and Man-Whitney U test were applied appropriate for sample distribution.

n	Infants with CHD	Controls	*p*
	77	44	
Male, n (%)	58 (75.3)	20 (45.5)	0.002
Birthweight, g (m (SD))	3347.7 (491.5)	3432.0 (402.5)	0.34
GA, weeks (m (SD))	39.4 (1.3)	39.6 (1.2)	0.42
Head circumference at birth, cm (m (SD))	34.6 (1.3)	35.1 (1.2)	0.035
Apgar 5 (M [IQR])	9.0 [8.0, 9.0]	9.0 [9.0, 9.0]	0.29
SES (2–12) (M [IQR])	8.0 [7.0, 10.0]	12.0 [10.0, 12.0]	<0.001
Cyanotic vitium, n (%)	69 (89.6)	NA	
Highest lactate preoperatively, mmol/l (M [IQR])	4.3 [3.1, 6.1]	NA	
Age at surgery, d (M [IQR])	12.0 [9.0, 16.0]	NA	
Extracorporeal circulation time, min (m (SD))	153.1 (78.0)	NA	
Aortic cross clamp time, min (m (SD))	93.4 (52.7)	NA	
Lowest temperature on bypass, °C (M [IQR])	28.1 [23.1, 31.5]	NA	
Days on ICU postoperatively (m (SD))	7.9 (10.1)	NA	

To control the false positive error rate for multiple comparisons, we subdivided our analyses according to the aims of the study into baseline analyses and confirmatory analyses. For baseline analyses, (including comparisons of baseline, clinical variables, brain volumes and neurodevelopmental outcomes between CHD infants and controls, and correlation analyses of risk factors with brain volume), p-values were considered significant at a level of <0.05 and no correction for multiple comparison was performed. In the confirmatory analyses, the multiple linear regression model testing the association of total brain volume with neurodevelopmental outcome was considered a global primary analysis and p-values < 0.05 were considered significant without p-value correction. For the remaining multiple regression analyses, testing the association of regional brain volumes with outcome, p-values were corrected by means of the Benjamini-Hochberg^29^ procedure with the *p.adjust* function from the *stats* package in R. The threshold for significance was set to 0.05 for the adjusted p-values; all unadjusted and adjusted p-values are provided.

## Results

### Study population

In this prospective cohort study, 77 infants with severe CHD were enrolled between December 2009 and August 2016 and 44 healthy infants were recruited as controls. In the CHD group, 70 infants had a preoperative MRI, 68 had a postoperative MRI and 60 had both a pre- and postoperative MRI. Reasons for missing preoperative brain MRI included patient instability and too short of a time period between recruitment and surgery (n = 7). In addition, 3 scans could not be included in the regional volumetric analyses due to motion artefacts. Postoperatively, 9 patients did not have a cerebral MRI, because infants were too old to undergo MRI in natural sleep (n = 6), or for logistical reasons. Furthermore, 6 postoperative scans could not be analysed due to poor image quality (see Supplementary Fig. S1). Among the MRIs of healthy controls, one could not be analysed due to motion artefacts. Hence, volumetric analyses were performed for 67 CHD infants preoperatively, 62 postoperatively and for 43 controls.

Table 1 presents demographic characteristics of the CHD infants and controls. Complete data (pre- and postoperative analysed brain scans and Bayley-III assessment) were collected for a total of 50 CHD infants. CHD diagnoses are listed in Table 2.

**Table 2:** Distribution of cardiac diagnoses of infants with CHD. CHD, congenital heart disease; dTGA, dextro-transposition of the great arteries; DORV, double outlet right ventricle; HLHS, hypoplastic left heart syndrome; HLHC, hypoplastic left heart complex.

Cardiac diagnosis	n
Biventricular	62
dTGA	38
Aortic arch anomaly	8
DORV TGA type	7
Other^†^	9
Univentricular	15
HLHS/HLHC	9
Other*	6

^†^Other biventricular diagnoses include neonates with atrioventricular septal defect (n = 2), ventricular septal defect (n = 1), pulmonary atresia with ventricular septal defect (n = 4), tetralogy of Fallot (n = 1) and levo-transposition of the great arteries (n = 1).

*Other univentricular diagnoses (non HLHS/HLHC) include neonates with tricuspid atresia (n = 3), pulmonary atresia with intact ventricular septum (n = 1), double inlet left ventricle (n = 1) and heterotaxy syndrome (n = 1).

### Surgical procedure

Cardiac surgery included biventricular repair by arterial switch or Rastelli operation for patients with dextro-transposition of the great arteries (dTGA), complex aortic arch surgery, systemic-pulmonary shunt procedure and early Fallot repair. For univentricular palliation, Norwood-type stage I palliation for patients with hypoplastic left heart syndrome and other forms of univentricular physiology with a hypoplastic aortic arch was performed^3^ (see Table 2 for CHD diagnoses). In the case of aortic arch surgery, i.e. Norwood repair, CPB surgery was performed under moderate-to-severe hypothermia (25 °C) with selective regional cerebral perfusion. Modified ultrafiltration was used at the end of CPB surgery. All patients were in a clinically stable state at the time of postoperative cerebral MRI and received medical treatment for postoperative congestive heart failure, including diuretics, beta blockers, and angiotensin converting enzyme inhibitors, as appropriate.

### Cerebral MRI

#### Time of MRI

In CHD infants, preoperative MRI was performed at a median [IQR] age of 7.0 [5.2, 8.8] days (40.2 [39.3, 41.2] postmenstrual weeks). Postoperative MRI was obtained at median 25.0 [20.8, 31.0] days of life (43.2 [41.4, 44.4] postmenstrual weeks) and 13.0 [10.0, 16.3] postoperative days. Controls were scanned at a median age of 21.0 [16.0, 28.0] days (42.3 [41.2, 43.6] postmenstrual weeks).

#### Pre- and postoperative brain injury

As assessed at preoperative MRI, 16 out of 70 (22.9%) patients had a brain lesion, comprised of either WMI (n = 14, 20%) and/or stroke (n = 4, 5.7%, 1 anterior cerebral artery, 2 middle cerebral artery, 1 posterior cerebral artery). Two infants had both WMI and stroke. At postoperative MRI new WMI was found in 2 of the 68 scanned infants (2.3%). No new strokes were observed. In 11 of the 14 infants with preoperative WMI, lesions persisted to postoperative scans (78.6%).

#### Volumetric findings

CHD infants had lower total and regional brain volumes in all regions pre- and postoperatively without a specific regional predilection (Table 3), compared to healthy controls. When comparing relative regional brain volumes (corrected for total brain volume) between CHD infants and controls we found the frontal lobe to be smaller in the CHD group preoperatively (ß = −0.18 95% CI −0.32 to −0.04, p = 0.012), whereas the parietal lobe appeared to be larger preoperatively (ß = 0.12, 95% CI 0.01 to 0.23, p = 0.035). For postoperative relative regional brain volumes, no difference was found. Additionally, total brain volume was found to be smaller in female compared to male CHD infants (preoperative: mean 347.27 cm^3^ vs. 379.53 cm^3^, 95% CI 4.26 to 60.25, p = 0.025; postoperative: 369.12 cm^3^ vs. 404.29 cm^3^, 95% CI 13.05 to 57.28, p = 0.002). In contrast, there was no difference in total brain volume between male and female controls (95% CI −50.13 to 13.35, p = 0.249).

**Table 3:** Pre- and postoperatively measured absolute brain volumes of CHD infants and controls in cm^3^ given as mean (standard deviation).

	Brain Volume			Group comparison	
	Preop n = 67	Postop n = 62	Controls n = 43	Preop vs. Controls, ß	Postop vs. Controls, ß
Total brain	371.3 (51.5)	395.2 (40.8)	434.7 (51.6)	−1.21^†^	−1.43^†^
Cortex	253.8 (43.5)	273.8 (33.7)	302.5 (39.4)	−1.06^†^	−1.29^†^
White matter	189.4 (26.7)	196.2 (20.7)	216.6 (26)	−0.81^†^	−0.97^†^
Frontal lobe	105.7 (13.4)	111.5 (11.3)	124.3 (14.9)	−0.72^†^	−0.82^†^
Parietal lobe	69.8 (11.3)	73.1 (8.6)	80.4 (11.8)	−0.45**	−0.59^†^
Occipital lobe	33.1 (6.9)	35.7 (4.1)	39.6 (4.7)	−0.43**	−0.44^†^
Temporal lobe	62.8 (8.8)	66.6 (7.2)	72.2 (8.8)	−0.46^†^	−0.51^†^
Cerebellum	31.1 (5.8)	33.9 (4.7)	37.7 (5.9)	−0.36**	−0.46^†^

P-values and ß coefficients (ß) based on multiple linear regression analyses adjusted for sex, postmenstrual age at MRI, MRI cohort. P-values coded as: **p < 0.001, ^†^p < 0.0001. Preop, preoperative; Postop, postoperative. Differences between pre- and postoperative brain volumes in CHD infants were significant, for details refer to Supplementary Table S2.

Preoperative and postoperative total brain volumes correlated with head circumference at birth (preoperative: *r* = 0.43, 95% CI 0.20 to 0.62, p = 0.0005; postoperative: *r* = 0.65, 95% CI 0.46 to 0.78, p < 0.0001), gestational age (preoperative: *r* = 0.29, 95% CI 0.05 to 0.5, p = 0.019; postoperative: *r* = 0.3, 95% CI 0.05 to 0.5, p = 0.018) and birth weight (preoperative: *r* = 0.47, 95% CI 0.25 to 0.63, p < 0.0001; postoperative: *r* = 0.57, 95% CI 0.37 to 0.71, p < 0.0001). Furthermore, we investigated the impact of perinatal and intraoperative variables (i. e. 5 min Apgar score, extracorporeal circulation time, lowest temperature on bypass, aortic cross clamp time) and found no significant correlation with postoperative total brain volumes (see Supplementary Table S1). However, a longer length of ICU stay was associated with smaller postoperative total brain volume (*r* = −0.25, 95% CI −0.48 to −0.00, p = 0.047). Further group comparisons revealed no difference in postoperative whole brain volume for infants with or without dTGA (mean 393.38 cm^3^ vs. 396.63 cm^3^, 95% CI −24.33 to 17.83, p = 0.53) or bi- versus univentricular heart defects (396.47 cm^3^ vs. 388.66 cm^3^, 95% CI −20.56 to 36.19, p = 0.76).

### Neurodevelopmental outcome at one year

Neurodevelopmental assessments were performed at a median [IQR] age of 12.0 months [12.0, 13.0] for CHD infants and 12.0 months [12.0, 12.0] for controls. One infant died prior to the one-year examination and 2 of 76 surviving CHD infants did not return for the neurodevelopmental assessment (follow up rate 97.4%). In two infants, the language score could not be assessed, as the infant was not exposed to or did not speak German as a main language. Among the 44 control children 7 were lost to follow up (follow up rate 84.1%). The Bayley-III CCS and MCS scores were significantly lower in CHD patients than in controls: CCS (mean (SD)) 105 (15.3) vs. 117.1 (11.3), 95% CI 6.43 to 17.68, p < 0.0001; MCS 92.7 (15.4) vs. 103.8 (10.9), 95% CI 5.50 to 16.74, p = 0.0002. For the LCS only a trend towards lower scores in CHD patients was found: 92.7 (14.0) vs. 97.5 (9.9), 95% CI −0.30 to 9.96, p = 0.06. After adjustment for SES, the differences in CCS (ß = −0.34, 95% CI −0.66 to −0.02, p = 0.037) and MCS (ß = −0.37, 95% CI −0.72 to −0.03, p = 0.036) among infants with CHD compared to controls remained, while no difference was found for the LCS (ß = −0.06, 95% CI −0.37 to 0.25, p = 0.70). Multiple linear regression, adjusted for SES revealed that MCS was higher for CHD patients with dTGA compared to patients with non-dTGA diagnoses (ß = 0.50, 95% CI 0.13 to 0.88, p = 0.009), while CCS and LCS did not differ between the cardiac diagnoses (CCS ß = 0.23, 95% CI −0.12 to 0.58, p = 0.19; LCS ß = −0.24, 95% CI −0.58 to 0.09, p = 0.15). The length of ICU stay was negatively associated with CCS (ß = −0.018, 95% CI −0.04 to −0.00, p = 0.045) and MCS (ß = −0.02, 95% CI −0.04 to −0.00, p = 0.036), but not with LCS (ß = −0.003, 95% CI −0.02 to 0.01, p = 0.73).

### Association between perioperative brain volumes and neurodevelopmental outcome

Preoperative brain volumes in CHD infants (including those from the total brain, cortex, white matter, frontal, parietal and temporal lobes and cerebellum) were not associated with one-year neurodevelopmental outcome when corrected for sex, postmenstrual age at time of MRI, MRI cohort and SES (see Supplementary Table S3). Similarly, no association was found between neonatal brain volume and one-year outcome in healthy controls (see Supplementary Table S4).

**Figure 2:**
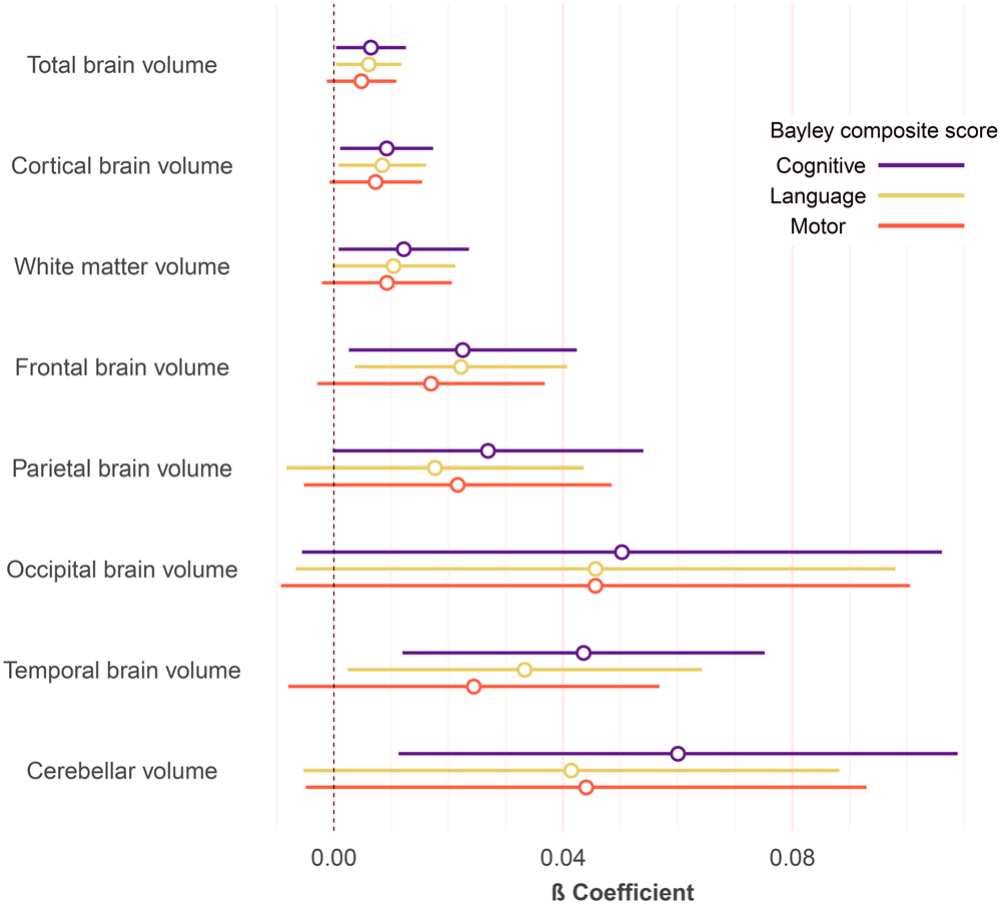
Association between postoperative total and regional brain volumes and one-year neurodevelopmental outcome in CHD infants. Dot-whisker plot of multiple linear regression models for each respective postoperative brain volume and neurodevelopmental outcome domain in CHD infants. Dots indicate the ß coefficients while whiskers display the 95% confidence interval. Models adjusted for sex, postmenstrual age at MRI, MRI cohort and socioeconomic status. Further model details and p-values adjusted for multiple comparison can be found in Supplementary Table S5.

In contrast, evidence was found that larger postoperative total and regional brain volumes were associated with better one-year neurodevelopmental outcome. Specifically, the total brain, cortical, white matter, frontal lobe, temporal lobe and cerebellar volumes were positively associated with CCS. Furthermore, total brain, cortical, frontal lobe and temporal lobe volumes were associated with LCS (Fig. 2), when adjusted for sex, postmenstrual age at time of MRI, MRI cohort and SES. When additionally introducing length of ICU stay into the multiple regression model, CCS remained associated with frontal (ß = 0.02, 95% CI 0.00 to 0.04, p = 0.043), temporal (ß = 0.039, 95% CI 0.01 to 0.07, p = 0.014) and cerebellar (ß = 0.056, 95% CI 0.01 to 0.10, p = 0.018) volumes, whereas LCS remained independently associated with cortical (ß = 0.0079, 95% CI 0.00 to 0.02, p = 0.045), temporal (ß = 0.032, 95% CI 0.00 to 0.06, p = 0.046) and frontal (ß = 0.021, 95% CI 0.00 to 0.04, p = 0.027) brain volumes. In contrast, relative regional volumes (adjusted for total brain volume) did not correlate with neurodevelopmental outcome either in the pre- or in the postoperative MRI in CHD infants and in controls. When multiple regression models, to test the association of regional brain volumes with outcome, were corrected for multiple comparisons, p-values did not remain significant at the < 0.05 threshold (Supplementary Table S5).

## Discussion

In this prospective cohort study, we examined the association between perioperative brain volumes and one-year neurodevelopmental outcome in children with severe CHD undergoing CPB surgery. In our cohort, CHD infants had smaller global and regional brain volumes than control infants both pre- and postoperatively. To the best of our knowledge, the present study is the first to show that postoperative total brain volume, as well as cortical, frontal, temporal, white matter and cerebellar volumes, were positively associated with neurodevelopmental outcome at one year. Preoperative brain volumes were not associated with outcome.

We found that brain volumes were reduced pre- and postoperatively in infants with CHD, and that all regional volumes were affected. This is in line with the finding that the majority of relative regional brain volumes (after correcting for total brain volume) were similar between CHD infants and controls. These results are in accordance with those by von Rhein *et al*.^3^ and Ortinau *et al*.^8^, and most likely reflect a general developmental disturbance of brain growth and connectivity of fetal origin^30^. Studies investigating older children with CHD showed that brain volume reduction persists for all types of CHD from early childhood^31–33^ until adolescence^34^. Importantly, we could not detect a trend towards isolated white or grey matter volume reduction in the CHD group. Watanabe *et al*. demonstrated a volume reduction that was most prominent in the grey matter, in particular in the frontal region, in children with CHD aged 13–15 months^31^. In contrast, Rollins *et al*. found a reduction in white matter volume^32^. However, in that study cerebral MRI was assessed at one year of age and alterations in brain growth may have become more apparent^32^. A study by Owen *et al*. demonstrated that the subcortical grey matter volume was reduced and the cerebrospinal fluid volume was increased in a mixed group of CHD^4^. Whether such a regional predilection (i.e. towards grey matter of certain lobes) is characteristic for CHD, however, would require further investigation, as the distribution of studied cardiac diagnoses and medical variables such as postoperative course and complications were often heterogeneous.

Although associations of brain volume reduction with functional outcome in CHD subjects were described for other age groups and follow up time points^4,32,35^, we are the first to demonstrate that neonatal total postoperative brain volume and certain regional volumes were positively associated with one-year cognitive and language outcome in CHD infants. While these associations were robustly observed for multiple brain regions and outcome domains, the strength of associations was weak, indicating that brain volume reductions do not fully account for the observed realm of neurodevelopmental variability in this cohort. After controlling for total brain volume, relative regional brain volumes did not correlate with outcome, as would be expected given the lack of a predilection for a certain brain region in the volume reduction.

While little is known about the association between neonatal brain volumes and outcome for the CHD populations, this association is well established for other at–risk populations. In children with perinatal asphyxia, hippocampal volume has been found to be reduced and associated with long-term outcome such as cognitive memory impairment as reviewed by de Haan *et al*.^36^. In preterm born infants, there is mounting evidence that reduced total and regional brain volumes predict neurodevelopmental outcome, particularly for the cerebellar volume^37–39^. However, in healthy infants in which no alteration or interruption of brain development occurs, an association between brain volume and outcome has not been found.

We further examined potential risk factors for reduced pre- and postoperative brain volumes and found that neonatal characteristics, such as head circumference at birth, gestational age and birth weight were related to larger pre- and postoperative total brain volume, while longer length of postoperative ICU stay was negatively associated with postoperative total brain volume. The length of stay in the ICU is typically an indicator of disease complexity and therefore appears to be associated with reduced brain volume and impaired neurodevelopmental outcome. In addition to this association, we found that, after adjusting for ICU stay, frontal, temporal and cerebellar brain volumes were independently associated with cognitive outcome, and cortical frontal and temporal volumes were associated with language outcome. This suggests that reduced brain volume reduction is not just a mediator of the negative effect of ICU stay on outcome, but may also be independently associated with outcome. The results are consistent with those of Rollins *et al*., who examined CHD children at one year of age and found an association between a longer ICU stay and reduced grey matter volume as well as between lower intraoperative pCO2 and reduced total brain volume. No other risk factors for lower brain volume were identified in their study^32^. By the same token, in our study, no surgical or cardiac factors such as extracorporeal circulation time, lowest temperature on bypass, aortic cross clamp time, univentricular CHD or dTGA were related to postoperative total brain volume. This observation is in line with the results from a large cohort of CHD infants in which measured intraoperative and postoperative factors accounted for less than 5% of the variances in one-year outcome, while patient-specific and preoperative factors, such as age or treatment centre contributed to outcome to almost 30%^40^.

Our study confirms that the predominant injuries in CHD infants are WMI and strokes, and new postoperative lesions are rare^2,10,11^. However, the prevalence of cerebral lesions detected at a given time may vary significantly depending on the type of CHD studied and timing of MRI^9–12,16,41^, due to possible longitudinal changes, such as the disappearance of white matter lesions on T1-weighted MRI. In our cohort the prevalence of cerebral lesions was relatively low. This is potentially a result of the rather late postoperative imaging time point and a selection bias towards infants who are clinically stable and can be scanned while in natural sleep. Therefore, the association of lesions with brain volumes and neurodevelopmental outcome was not analysed, and the injury status was not included in our multiple regression models, although others have demonstrated a correlation between perioperative brain injuries and early childhood outcome^12,16,17^.

Our outcomes are in line with the findings of Andropoulos *et al*. who also used the Bayley-III assessment in their cohort^16^: Neurodevelopmental outcome in the CHD population was lower in all domains, but within the normal range. One possible interpretation of this finding is that the Bayley-III generates significantly higher scores than the Bayley-II, and thus might overestimate functional outcome^42^. Therefore, test version needs to be considered when comparing outcome results among studies.

Our findings provide further evidence for a “multiple-hit” theory, which could explain why the preoperatively measured brain volumes did not correlate with outcome, whereas the postoperative volumes did. In this model, the “first hit” takes place during prenatal life, caused by different factors such as hypoperfusion and neuronal disturbances, while the second hit occurs during the perioperative phase. The combined effect of these events may only become evident later in life, in addition to an increased inter-individual variability of brain growth rates. A more precise assessment of a possible divergence in such growth trajectories would require longitudinal neurodevelopmental evaluations and repeated MR imaging with volumetry at multiple time points after surgery.

This study has several strengths. Pre- and postoperative MRIs could be obtained in a large number of patients and brain volumes were measured with an automated pipeline in a fast and reliable fashion. The follow up rate at one-year was excellent and assessment was performed with a detailed, standardized developmental test (Bayley-III). However, it is important to note that the predictive value of the Bayley-III assessment for long-term neurodevelopmental outcome is limited^43^. Later neurodevelopmental follow up is warranted to confirm our results. Another limitation of the study is that, our study population is a heterogeneous, clinically diverse sample composed of patients with various CHD diagnoses. Subgroup analyses or stratification of the linear regression results by the CHD diagnoses would yield more specific results, but this leads to a reduced sample size, thereby also reducing the power to detect differences. Additionally, there is a potential inflation of the type I error rate, as many regression analyses were performed, to investigate the association of regional brain volumes with neurodevelopmental outcome. This resulted in multiple hypothesis tests being performed. We thus corrected all p-values of the confirmatory analyses, testing the association of regional brain volumes with outcome; all adjusted p-values are provided in the Supplement. However, after adjustment for multiple comparisons, the p-values did not remain significant, indicating that additional dedicated studies with a larger sample size are needed to confirm our results. A technical limitation of our study is that the MRI scanner was upgraded during the study. While the same pulse sequence was used, the upgrade could have theoretically affected the MRI contrast, potentially introducing a bias into the brain volumes calculated using the automated approach. To control for the confounding effect of MRI scanner software version, we corrected for this variable in all statistical analyses, and we additionally validated the volumes derived using the automated approach with those from a manual gold-standard method. However, the in-house developed automatic work-flow should also be compared to alternative automated approaches in the future^27^. During the validation of the anatomical prior based automatic segmentation approach (Supplementary Note), we found high variability for total cortical volume, which limits the generalisability of our volumetric findings. More reliable and reproducible results can be expected for lobar white matter, total brain volume and cerebellar volume estimates, which was confirmed by higher correlation and spatial overlap of these volume estimates with a more recently published automated approach (dHCP pipeline, more details are found in Supplementary Note), and higher correlation to manual stereology.

In conclusion, we found that pre- and postoperative total and regional brain volumes of children with CHD were smaller when compared to controls. Smaller postoperative brain volumes were weakly associated with poorer neurodevelopmental outcome while preoperative brain volumes were not. Our findings provide further support for an association between neonatal brain volumes and neurodevelopmental outcome across at-risk populations. Our results underscore the importance of early neuroimaging assessments in infants with severe CHD, which provides measures such as the total brain volume which could serve as a marker for altered brain development.

## Supplementary information

Supplementary information

## Data Availability

The datasets generated and analysed during the current study are available from the corresponding author on reasonable request.
