# Supplementary material for: Postoperative brain volumes are associated with one-year neurodevelopmental outcome in children with severe congenital heart disease
Source: Sci Rep. 2019 Jul 26;9:10885. doi: 10.1038/s41598-019-47328-9 (PMC6659678; doi:10.1038/s41598-019-47328-9)
Supplement: Supplementary file 1 — Supplementary information [file 41598_2019_47328_MOESM1_ESM.docx]

**Supplementary Information**

**Postoperative brain volumes are associated with one-year neurodevelopmental outcome in children with severe congenital heart disease**

Eliane Meuwly and Maria Feldmann, Walter Knirsch, Michael von Rhein, Kelly Payette, Hitendu Dave, Ruth O’Gorman Tuura, Raimund Kottke, Cornelia Hagmann, Beatrice Latal and András Jakab, on behalf of the Heart and Brain Research Group

**Supplementary Figure S1**. **Flowchart of enrolled CHD infants.** CHD, congenital heart disease; postop, postoperative; preop, preoperative; MRI, magnetic resonance imaging, Bayley-III, Bayley Scales of Infant and Toddler Development, Third Edition.

**Supplementary Table S1.** **Correlation between perinatal and intraoperative risk factors and postoperative total brain volume.** Pearson correlation of risk factors and postoperative total brain volume. *r*, correlation coefficient; CI, confidence interval; ECC, extracorporeal circulation time; ACC, aortic cross clamp time.

| **Risk factor** | ***r*** | **95 % CI** | ***p*** |
| --- | --- | --- | --- |
| Apgar 5 min | -0.017 | -0.26; 0.23 | 0.90 |
| ECC | 0.072 | -0.19; 0.32 | 0.58 |
| Lowest temperature on bypass | 0.14 | -0.13; 0.38 | 0.32 |
| ACC | 0.079 | -0.18; 0.32 | 0.55 |

**Supplementary Table S2.** **Comparison of pre- and postoperative brain volumes in CHD infants.** Paired t test. CI, confidence interval.

| Brain volume | Mean difference (cm^3^) | 95 % CI | *p* |
| --- | --- | --- | --- |
| Total brain volume | 30.49 | 18.42; 42.56 | < 0.0001 |
| Cortex | 25.97 | 15.91; 36.03 | < 0.0001 |
| White matter | 10.45 | 4.29; 16.60 | 0.0013 |
| Frontal lobe | 7.67 | 4.36; 10.98 | < 0.0001 |
| Parietal lobe | 4.55 | 2.16; 6.94 | 0.0003 |
| Occipital lobe | 3.61 | 1.87; 5.35 | 0.0001 |
| Temporal lobe | 5.10 | 3.28; 6.93 | < 0.0001 |
| Cerebellum | 3.94 | 2.74; 5.14 | < 0.0001 |

**Supplementary Table S3.** **Association between preoperative neonatal brain volume and one-year neurodevelopmental outcome in CHD infants.** Multiple linear regression models for each respective preoperative brain volume and neurodevelopmental outcome domain in CHD infants. ß, ß coefficient of the covariates; CI, confidence interval; Adj, adjusted; TBV, total brain volume; PMA, postmenstrual age; MRI, magnetic resonance imaging; SES, socioeconomic status. p Model corresponds to p-value of the linear regression model.

|  | **Cognitive Composite Score** | | | **Language Composite Score** | | | **Motor Composite Score** | | |
| --- | --- | --- | --- | --- | --- | --- | --- | --- | --- |
|  | **ß** | **95 % CI** | ***p*** | **ß** | **95 % CI** | ***p*** | **ß** | **95 % CI** | ***p*** |
| Intercept | 9,52 | 5.60 ; 13.44 | < 0.0001 | 8,34 | 4.61 ; 12.06 | < 0.0001 | 7,3 | 2.99 ; 11.62 | 0.001 |
| **TBV** | 0,0038 | -0.00 ; 0.01 | 0,13 | 0,0032 | -0.00 ; 0.01 | 0,17 | 0,00068 | -0.00 ; 0.01 | 0,8 |
| Sex | -0,33 | -0.77 ; 0.11 | 0,14 | -0,23 | -0.65 ; 0.18 | 0,27 | -0,11 | -0.59 ; 0.38 | 0,66 |
| PMA at MRI | -0.028 | -0.12 ; 0.06 | 0,53 | -0.016 | -0.10 ; 0.07 | 0,71 | 0.031 | -0.07 ; 0.13 | 0,53 |
| MRI Cohort | 0,2 | -0.18 ; 0.58 | 0,29 | 0,24 | -0.12 ; 0.60 | 0,19 | 0,41 | -0.01 ; 0.83 | 0.055 |
| SES | 0.091 | 0.00 ; 0.18 | 0.045 | 0,1 | 0.02 ; 0.18 | 0,02 | 0.068 | -0.03 ; 0.17 | 0,17 |
|  | Adj R^2^: 0.073 | | | Adj R^2^: 0.091 | | | Adj R^2^: 0.027 | | |
|  | *p* Model: 0.12 | | | *p* Model: 0.081 | | | *p* Model: 0.28 | | |
| Intercept | 10,1 | 6.27 ; 13.94 | < 0.0001 | 8,84 | 5.18 ; 12.49 | < 0.0001 | 7,43 | 3.15 ; 11.72 | 0.001 |
| **Cortex** | 0,0063 | 0.00 ; 0.01 | 0.047 | 0,0054 | -0.00 ; 0.01 | 0.073 | 0,0018 | -0.01 ; 0.01 | 0,61 |
| Sex | -0,38 | -0.81 ; 0.06 | 0.089 | -0,27 | -0.69 ; 0.14 | 0,19 | -0,13 | -0.62 ; 0.36 | 0,6 |
| *P*MA at MRI | -0.044 | -0.14 ; 0.05 | 0,34 | -0,03 | -0.12 ; 0.06 | 0,5 | 0.025 | -0.08 ; 0.13 | 0,63 |
| MRI Cohort | 0,1 | -0.29 ; 0.49 | 0,6 | 0,15 | -0.22 ; 0.53 | 0,41 | 0,38 | -0.06 ; 0.81 | 0.088 |
| SES | 0.092 | 0.00 ; 0.18 | 0.039 | 0,1 | 0.02 ; 0.18 | 0.017 | 0.068 | -0.03 ; 0.16 | 0,17 |
|  | Adj R^2^: 0.10 | | | Adj R^2^: 0.12 | | | Adj R^2^: 0.031 | | |
|  | *p* Model: 0.061 | | | *p* Model: 0.047 | | | *p* Model: 0.26 | | |
| Intercept | 9,18 | 5.12 ; 13.25 | < 0.0001 | 8,05 | 4.19 ; 11.91 | 0,0001 | 7,45 | 3.01 ; 11.89 | 0.001 |
| **White matter** | 0.006 | -0.00 ; 0.02 | 0,21 | 0,0051 | -0.00 ; 0.01 | 0,25 | -0,00072 | -0.01 ; 0.01 | 0,89 |
| Sex | -0,3 | -0.74 ; 0.13 | 0,17 | -0,21 | -0.62 ; 0.20 | 0,31 | -0.082 | -0.56 ; 0.40 | 0,73 |
| PMA at MRI | -0.016 | -0.10 ; 0.07 | 0,71 | -0,0055 | -0.09 ; 0.08 | 0,9 | 0.036 | -0.06 ; 0.13 | 0,45 |
| MRI Cohort | 0,29 | -0.11 ; 0.68 | 0,15 | 0,31 | -0.06 ; 0.69 | 0,1 | 0,4 | -0.03 ; 0.84 | 0.065 |
| SES | 0.091 | 0.00 ; 0.18 | 0.046 | 0,1 | 0.02 ; 0.19 | 0.021 | 0,07 | -0.03 ; 0.17 | 0,16 |
|  | Adj R^2^: 0.061 | | | Adj R^2^: 0.080 | | | Adj R^2^: 0.026 | | |
|  | *p* Model: 0.15 | | | *p* Model: 0.10 | | | *p* Model: 0.28 | | |
| Intercept | 9,49 | 5.56 ; 13.42 | < 0.0001 | 8,24 | 4.54 ; 11.94 | < 0.0001 | 7,44 | 3.12 ; 11.76 | 0.001 |
| **Frontal lobe** | 0.014 | -0.00 ; 0.03 | 0,14 | 0.014 | -0.00 ; 0.03 | 0,1 | -0,0028 | -0.02 ; 0.02 | 0,79 |
| Sex | -0,34 | -0.79 ; 0.10 | 0,13 | -0,26 | -0.68 ; 0.16 | 0,22 | -0.069 | -0.56 ; 0.42 | 0,78 |
| PMA at MRI | -0.029 | -0.12 ; 0.06 | 0,52 | -0.021 | -0.11 ; 0.06 | 0,63 | 0,04 | -0.06 ; 0.14 | 0,43 |
| MRI Cohort | 0,25 | -0.13 ; 0.63 | 0,19 | 0,29 | -0.07 ; 0.65 | 0,11 | 0,41 | -0.01 ; 0.83 | 0.056 |
| SES | 0.089 | -0.00 ; 0.18 | 0.051 | 0.097 | 0.01 ; 0.18 | 0.024 | 0.071 | -0.03 ; 0.17 | 0,15 |
|  | Adj R^2^: 0.071 | | | Adj R^2^: 0.10 | | | Adj R^2^: 0.027 | | |
|  | *p* Model: 0.12 | | | *p* Model: 0.060 | | | *p* Model: 0.27 | | |
| Intercept | 9,3 | 5.43 ; 13.17 | < 0.0001 | 8,39 | 4.61 ; 12.17 | < 0.0001 | 7,15 | 2.84 ; 11.46 | 0.002 |
| **Parietal lobe** | 0.022 | 0.00 ; 0.04 | 0.046 | 0,0095 | -0.01 ; 0.03 | 0,38 | 0,0086 | -0.02 ; 0.03 | 0,48 |
| Sex | -0,33 | -0.75 ; 0.09 | 0,12 | -0,19 | -0.61 ; 0.22 | 0,35 | -0,12 | -0.60 ; 0.35 | 0,6 |
| PMA at MRI | -0.028 | -0.12 ; 0.06 | 0,52 | -0.007 | -0.09 ; 0.08 | 0,87 | 0.027 | -0.07 ; 0.12 | 0,57 |
| MRI Cohort | 0,29 | -0.09 ; 0.67 | 0,13 | 0,29 | -0.08 ; 0.65 | 0,13 | 0,44 | 0.02 ; 0.86 | 0.042 |
| SES | 0.093 | 0.01 ; 0.18 | 0.037 | 0,1 | 0.02 ; 0.19 | 0.018 | 0.068 | -0.03 ; 0.16 | 0,17 |
|  | Adj R^2^: 0.10 | | | Adj R^2^: 0.071 | | | Adj R^2^: 0.036 | | |
|  | *p* Model: 0.06 | | | *p* Model: 0.12 | | | *p* Model: 0.24 | | |
| Intercept | 9,78 | 5.78 ; 13.77 | < 0.0001 | 8,48 | 4.72 ; 12.25 | < 0.0001 | 7,37 | 3.07 ; 11.68 | 0.001 |
| **Occipital lobe** | 0,0078 | -0.03 ; 0.04 | 0,66 | 0.014 | -0.02 ; 0.05 | 0,41 | -0,00057 | -0.04 ; 0.04 | 0,98 |
| Sex | -0,26 | -0.70 ; 0.18 | 0,24 | -0,18 | -0.60 ; 0.23 | 0,37 | -0.088 | -0.56 ; 0.38 | 0,71 |
| PMA at MRI | -0.011 | -0.10 ; 0.08 | 0,81 | -0,0038 | -0.09 ; 0.08 | 0,93 | 0.035 | -0.06 ; 0.13 | 0,46 |
| MRI Cohort | 0,21 | -0.18 ; 0.60 | 0,28 | 0,24 | -0.13 ; 0.60 | 0,2 | 0,41 | -0.01 ; 0.83 | 0.054 |
| SES | 0.095 | 0.00 ; 0.19 | 0.039 | 0,1 | 0.02 ; 0.19 | 0.018 | 0.069 | -0.03 ; 0.17 | 0,16 |
|  | Adj R^2^: 0.034 | | | Adj R^2^: 0.069 | | | Adj R^2^: 0.026 | | |
|  | *p* Model: 0.25 | | | *p* Model: 0.13 | | | *p* Model: 0.28 | | |
| Intercept | 9,32 | 5.42 ; 13.21 | < 0.0001 | 8,14 | 4.44 ; 11.84 | < 0.0001 | 7,34 | 3.01 ; 11.68 | 0.001 |
| **Temporal lobe** | 0.027 | -0.00 ; 0.06 | 0.068 | 0.024 | -0.00 ; 0.05 | 0.082 | 0,0012 | -0.03 ; 0.03 | 0,94 |
| Sex | -0,37 | -0.81 ; 0.07 | 0.098 | -0,27 | -0.69 ; 0.15 | 0,2 | -0.096 | -0.59 ; 0.39 | 0,7 |
| PMA at MRI | -0.029 | -0.12 ; 0.06 | 0,52 | -0.017 | -0.10 ; 0.07 | 0,68 | 0.034 | -0.06 ; 0.13 | 0,48 |
| MRI Cohort | 0,22 | -0.15 ; 0.60 | 0,24 | 0,26 | -0.10 ; 0.61 | 0,15 | 0,41 | -0.00 ; 0.83 | 0.052 |
| SES | 0.087 | -0.00 ; 0.18 | 0.052 | 0.097 | 0.01 ; 0.18 | 0.024 | 0.069 | -0.03 ; 0.17 | 0,16 |
|  | Adj R^2^: 0.093 | | | Adj R^2^: 0.11 | | | Adj R^2^: 0.026 | | |
|  | *p* Model: 0.077 | | | *p* Model: 0.051 | | | *p* Model: 0.28 | | |
| Intercept | 9,91 | 6.03 ; 13.79 | < 0.0001 | 8,65 | 4.90 ; 12.40 | < 0.0001 | 7,39 | 3.14 ; 11.65 | 0.001 |
| **Cerebellum** | 0,04 | -0.01 ; 0.09 | 0.099 | 0.017 | -0.03 ; 0.06 | 0,47 | 0.022 | -0.03 ; 0.07 | 0,4 |
| Sex | -0,29 | -0.71 ; 0.14 | 0,18 | -0,17 | -0.58 ; 0.24 | 0,4 | -0,11 | -0.58 ; 0.35 | 0,62 |
| PMA at MRI | -0.037 | -0.13 ; 0.06 | 0,42 | -0,01 | -0.10 ; 0.08 | 0,82 | 0.019 | -0.08 ; 0.12 | 0,71 |
| MRI Cohort | 0,16 | -0.22 ; 0.55 | 0,4 | 0,23 | -0.14 ; 0.60 | 0,22 | 0,38 | -0.04 ; 0.80 | 0.078 |
| SES | 0,11 | 0.02 ; 0.20 | 0.019 | 0,11 | 0.02 ; 0.20 | 0.013 | 0.075 | -0.02 ; 0.17 | 0,13 |
|  | Adj R^2^: 0.082 | | | Adj R^2^: 0.066 | | | Adj R^2^: 0.04 | | |
|  | *p* Model: 0.097 | | | *p* Model: 0.14 | | | *p* Model: 0.22 | | |

**Supplementary Table S4.** **Association between neonatal brain volume and one-year neurodevelopmental outcome in healthy controls**. Multiple linear regression models for total brain volume and each neurodevelopmental outcome domain in healthy controls. ß, ß coefficient of the covariates; CI, confidence interval; Adj, adjusted; TBV, total brain volume; PMA, postmenstrual age; MRI, magnetic resonance imaging; SES, socioeconomic status. p Model corresponds to p-value of the linear regression model.

|  | **Cognitive Composite Score** | | | **Language Composite Score** | | | **Motor Composite Score** | | |
| --- | --- | --- | --- | --- | --- | --- | --- | --- | --- |
|  | **ß** | **95 % CI** | ***p*** | **ß** | **95 % CI** | ***p*** | **ß** | **95 % CI** | ***p*** |
| Intercept | 8,47 | 1.68; 15.26 | 0.016 | 8,35 | 1.50; 15.20 | 0.019 | 9 | 1.65; 16.36 | 0.018 |
| **TBV** | 0,0045 | -0.00 ; 0.01 | 0,2 | -0,00084 | -0.01 ; 0.01 | 0,81 | 0,0014 | -0.01 ; 0.01 | 0,71 |
| Sex | -0,26 | -0.70 ; 0.18 | 0,24 | 0,23 | -0.22 ; 0.67 | 0,3 | -0.053 | -0.53 ; 0.43 | 0,82 |
| PMA at MRI | 0,0077 | -0.21 ; 0.22 | 0,94 | 0.051 | -0.17 ; 0.27 | 0,63 | -0,0047 | -0.24 ; 0.23 | 0,97 |
| MRI Cohort | -0,26 | -0.67 ; 0.15 | 0,21 | 0,23 | -0.18 ; 0.65 | 0,25 | 0,2 | -0.24 ; 0.64 | 0,36 |
| SES | 0.029 | -0.11 ; 0.17 | 0,67 | -0.048 | -0.19 ; 0.09 | 0,48 | 0.062 | -0.09 ; 0.21 | 0,4 |
|  | Adj R^2^: 0.096 | | | Adj R^2^: -0.077 | | | Adj R^2^: -0.091 | | |
|  | *p* Model: 0.18 | | | *p* Model: 0.73 | | | *p* Model: 0.79 | | |

**Supplementary Table S5.** **Association between postoperative neonatal brain volume and one-year neurodevelopmental outcome in CHD infants.** Multiple linear regression models for each respective postoperative regional brain volume and neurodevelopmental outcome domain in CHD infants. ß, ß coefficient of the covariates; CI, confidence interval; Adj, adjusted; TBV, total brain volume; PMA, postmenstrual age; MRI, magnetic resonance imaging; SES, socioeconomic status. p Model corresponds to p-value of the linear regression model. P values of brain volumes independently associated with outcome, unadjusted for multiple comparison, are printed in bold. P-values of associations of regional brain volumes with outcome were adjusted for multiple comparison and were annotated with *.

|  | **Cognitive Composite Score** | | | **Language Composite Score** | | | **Motor Composite Score** | | |
| --- | --- | --- | --- | --- | --- | --- | --- | --- | --- |
|  | **ß** | **95% CI** | ***p*** | **ß** | **95% CI** | ***p*** | **ß** | **95% CI** | ***p*** |
| Intercept | 7,33 | 3.35 ; 11.31 | 0,0005 | 7,62 | 3.91 ; 11.32 | 0,0001 | 3,94 | -0.02 ; 7.90 | 0.051 |
| **TBV** | 0,0065 | 0.00 ; 0.01 | **0.037** | 0,0061 | 0.00 ; 0.01 | **0.037** | 0,0048 | -0.00 ; 0.01 | 0,12 |
| Sex | -0,33 | -0.85 ; 0.18 | 0,2 | -0,34 | -0.82 ; 0.15 | 0,17 | -0,16 | -0.67 ; 0.36 | 0,54 |
| PMA at MRI | -0,0046 | -0.11 ; 0.10 | 0,93 | -0,03 | -0.13 ; 0.07 | 0,54 | 0.069 | -0.03 ; 0.17 | 0,18 |
| MRI Cohort | -0.022 | -0.42 ; 0.38 | 0,91 | 0,2 | -0.18 ; 0.57 | 0,3 | 0,26 | -0.14 ; 0.65 | 0,2 |
| SES | 0.095 | 0.00 ; 0.19 | 0.049 | 0,12 | 0.03 ; 0.21 | 0.013 | 0.085 | -0.01 ; 0.18 | 0.076 |
|  | Adj. R^2^: 0.11 | | | Adj R^2^: 0.15 | | | Adj R^2^: 0.15 | | |
|  | *p* Model: 0.049 | |  | *p* Model: 0.017 | |  | *p* Model: 0.018 | |  |
| Intercept | 8,47 | 4.34 ; 12.61 | 0,0001 | 8,66 | 4.80 ; 12.52 | < 0.0001 | 4,86 | 0.74 ; 8.97 | 0.022 |
| **Cortex** | 0,0092 | 0.00 ; 0.02 | **0.027*** | 0,0084 | 0.00 ; 0.02 | **0.031*** | 0,0073 | -0.00 ; 0.02 | 0.075* |
| Sex | -0,34 | -0.84 ; 0.17 | 0,19 | -0,33 | -0.81 ; 0.14 | 0,17 | -0,17 | -0.67 ; 0.33 | 0,5 |
| PMA at MRI | -0.028 | -0.14 ; 0.08 | 0,62 | -0,05 | -0.16 ; 0.06 | 0,34 | 0.047 | -0.06 ; 0.16 | 0,4 |
| MRI Cohort | -0,14 | -0.57 ; 0.28 | 0,5 | 0.085 | -0.32 ; 0.49 | 0,67 | 0,16 | -0.26 ; 0.58 | 0,46 |
| SES | 0.095 | 0.00 ; 0.19 | 0.049 | 0,12 | 0.03 ; 0.21 | 0.011 | 0.084 | -0.01 ; 0.18 | 0.078 |
|  | Adj R^2^: 0.12 | | | Adj R^2^: 0.16 | | | Adj R^2^: 0.16 | | |
|  | *p* Model: 0.039 | | *****0.094 | *p* Model: 0.015 | | *****0.094 | *p* Model: 0.013 | | *****0.12 |
| Intercept | 5,79 | 1.64 ; 9.94 | 0.007 | 6,26 | 2.37 ; 10.16 | 0.002 | 2,77 | -1.36 ; 6.90 | 0,18 |
| **White matter** | 0.012 | 0.00 ; 0.02 | **0.036*** | 0,01 | -0.00 ; 0.02 | 0.057* | 0,0093 | -0.00 ; 0.02 | 0,11* |
| Sex | -0,31 | -0.81 ; 0.19 | 0,23 | -0,3 | -0.78 ; 0.18 | 0,21 | -0,14 | -0.63 ; 0.36 | 0,58 |
| PMA at MRI | 0.033 | -0.06 ; 0.12 | 0,46 | 0,0082 | -0.08 ; 0.09 | 0,85 | 0.097 | 0.01 ; 0.19 | 0.035 |
| MRI Cohort | 0,18 | -0.24 ; 0.60 | 0,39 | 0,38 | -0.02 ; 0.78 | 0,06 | 0,41 | -0.01 ; 0.83 | 0.055 |
| SES | 0.089 | -0.01 ; 0.18 | 0.068 | 0,11 | 0.02 ; 0.21 | 0.019 | 0,08 | -0.02 ; 0.18 | 0.097 |
|  | Adj R^2^: 0.11 | | | Adj R^2^: 0.14 | | | Adj R^2^: 0.15 | | |
|  | *p* Model: 0.047 *****0.094 | | | *p* Model: 0.023 *****0.12 | | | *p* Model: 0.017 *****0.12 | | |
| Intercept | 6,25 | 2.23 ; 10.27 | 0.003 | 6,56 | 2.83 ; 10.28 | 0,0009 | 3,13 | -0.88 ; 7.14 | 0,12 |
| **Frontal lobe** | 0.022 | 0.00 ; 0.04 | **0.027*** | 0.022 | 0.00 ; 0.04 | **0,02*** | 0.017 | -0.00 ; 0.04 | 0.092* |
| Sex | -0,31 | -0.81 ; 0.18 | 0,21 | -0,33 | -0.80 ; 0.13 | 0,16 | -0,14 | -0.63 ; 0.35 | 0,57 |
| PMA at MRI | 0.021 | -0.07 ; 0.11 | 0,65 | -0,0067 | -0.09 ; 0.08 | 0,88 | 0.088 | -0.00 ; 0.18 | 0.062 |
| MRI Cohort | 0.048 | -0.35 ; 0.44 | 0,81 | 0,27 | -0.10 ; 0.64 | 0,15 | 0,31 | -0.09 ; 0.70 | 0,12 |
| SES | 0.093 | -0.00 ; 0.19 | 0.053 | 0,11 | 0.02 ; 0.20 | 0.015 | 0.084 | -0.01 ; 0.18 | 0.081 |
|  | Adj R^2^: 0.12 | | | Adj R^2^: 0.17 | | | Adj R^2^: 0.15 | | |
|  | *p* Model: 0.039 | | *****0.094 | *p* Model: 0.011 | | *****0.094 | *p* Model: 0.015 | | *****0.12 |
| Intercept | 6,63 | 2.61 ; 10.65 | 0.002 | 7,08 | 3.27 ; 10.90 | 0,0005 | 3,39 | -0.59 ; 7.37 | 0.094 |
| **Parietal lobe** | 0.027 | -0.00 ; 0.05 | 0.052* | 0.018 | -0.01 ; 0.04 | 0,18* | 0.022 | -0.01 ; 0.05 | 0,11* |
| Sex | -0,28 | -0.77 ; 0.22 | 0,27 | -0,23 | -0.71 ; 0.25 | 0,34 | -0,12 | -0.62 ; 0.37 | 0,61 |
| PMA at MRI | 0.023 | -0.07 ; 0.12 | 0,63 | 0,0047 | -0.08 ; 0.09 | 0,92 | 0.088 | -0.01 ; 0.18 | 0.064 |
| MRI Cohort | 0,14 | -0.28 ; 0.55 | 0,51 | 0,32 | -0.08 ; 0.72 | 0,11 | 0,38 | -0.03 ; 0.79 | 0.068 |
| SES | 0.094 | -0.00 ; 0.19 | 0.053 | 0,12 | 0.03 ; 0.22 | 0.012 | 0.084 | -0.01 ; 0.18 | 0.082 |
|  | Adj R^2^: 0.097 | | | Adj R^2^: 0.11 | | | Adj R^2^: 0.15 | | |
|  | *p* Model: 0.061 | | *****0.12 | *p* Model: 0.048 | | *****0.18 | *p* Model: 0.017 | | *****0.12 |
| Intercept | 7,2 | 3.19 ; 11.22 | 0,0007 | 7,5 | 3.75 ; 11.25 | 0,0002 | 3,86 | -0.09 ; 7.81 | 0.055 |
| **Occipital lobe** | 0,05 | -0.01 ; 0.11 | 0.077* | 0.046 | -0.01 ; 0.10 | 0.086* | 0.046 | -0.01 ; 0.10 | 0,1* |
| Sex | -0,22 | -0.70 ; 0.27 | 0,37 | -0,23 | -0.69 ; 0.23 | 0,32 | -0.093 | -0.57 ; 0.38 | 0,7 |
| PMA at MRI | 0.015 | -0.08 ; 0.11 | 0,77 | -0.011 | -0.10 ; 0.08 | 0,82 | 0.077 | -0.02 ; 0.17 | 0,12 |
| MRI Cohort | 0,0023 | -0.40 ; 0.40 | 0,99 | 0,22 | -0.16 ; 0.60 | 0,25 | 0,27 | -0.12 ; 0.67 | 0,18 |
| SES | 0.091 | -0.01 ; 0.19 | 0.068 | 0,12 | 0.02 ; 0.21 | 0.017 | 0.078 | -0.02 ; 0.17 | 0,11 |
|  | Adj R^2^: 0.086 | | | Adj R^2^: 0.13 | | | Adj R^2^: 0.15 | | |
|  | *p* Model: 0.078 | | *****0.12 | *p* Model: 0.031 | | *****0.12 | *p* Model: 0.016 | | *****0.12 |
| Intercept | 6.87 | 3.00 ; 10.75 | 0.0008 | 7.21 | 3.51 ; 10.91 | 0.0003 | 3.64 | -0.33 ; 7.60 | 0.072 |
| **Temporal lobe** | 0.044 | 0.01 ; 0.08 | **0.008*** | 0.033 | 0.00 ; 0.06 | **0.035*** | 0.024 | -0.01 ; 0.06 | 0.14* |
| Sex | -0.39 | -0.89 ; 0.10 | 0.12 | -0.33 | -0.81 ; 0.15 | 0.17 | -0.14 | -0.65 ; 0.37 | 0.59 |
| PMA at MRI | -0.00061 | -0.10 ; 0.09 | 0.99 | -0.016 | -0.11 ; 0.07 | 0.73 | 0.082 | -0.02 ; 0.18 | 0.096 |
| MRI Cohort | 0.046 | -0.34 ; 0.43 | 0.81 | 0.27 | -0.11 ; 0.64 | 0.16 | 0.3 | -0.09 ; 0.70 | 0.13 |
| SES | 0.09 | -0.00 ; 0.18 | 0.058 | 0.11 | 0.02 ; 0.20 | 0.017 | 0.085 | -0.01 ; 0.18 | 0.077 |
|  | Adj R^2^: 0.15 | | | Adj R^2^: 0.15 | | | Adj R^2^: 0.14 | | |
|  | *p* Model: 0.016 | | *****0.094 | *p* Model: 0.016 | | *****0.094 | *p* Model: 0.020 | | *****0.14 |
| Intercept | 7,98 | 4.00 ; 11.97 | 0,0002 | 8 | 4.19 ; 11.81 | < 0.0001 | 4,41 | 0.42 ; 8.41 | 0.031 |
| **Cerebellum** | 0,06 | 0.01 ; 0.11 | **0.017*** | 0.041 | -0.01 ; 0.09 | 0.081* | 0.044 | -0.00 ; 0.09 | 0.077* |
| Sex | -0,24 | -0.70 ; 0.22 | 0,31 | -0,23 | -0.68 ; 0.23 | 0,33 | -0.083 | -0.55 ; 0.38 | 0,72 |
| PMA at MRI | -0.011 | -0.11 ; 0.09 | 0,83 | -0,02 | -0.12 ; 0.08 | 0,69 | 0.065 | -0.04 ; 0.17 | 0,2 |
| MRI Cohort | -0.052 | -0.45 ; 0.34 | 0,79 | 0,2 | -0.19 ; 0.58 | 0,31 | 0,23 | -0.16 ; 0.63 | 0,24 |
| SES | 0,11 | 0.01 ; 0.20 | 0.026 | 0,13 | 0.04 ; 0.22 | 0.006 | 0.093 | 0.00 ; 0.19 | 0.048 |
|  | Adj R^2^: 0.13 | | | Adj R^2^: 0.13 | | | Adj R^2^: 0.16 | | |
|  | *p* Model: 0.028 | | *****0.94 | *p* Model: 0.029 | | *****0.12 | *p* Model: 0.013 | | *****0.12 |

**Supplementary Note**

**Validation of the atlas prior based volumetry method**

***Patient population***

We validated two approaches for automatic brain volumetry: atlas prior based estimation (unpublished method used for testing the main hypothesis of this manuscript) and an expectation maximization based algorithm, implemented in the processing pipeline of the developing human connectome project dHCP (Makropoulos et al., 2014). Three different, overlapping subsets of the total study population were used for validation (Supplementary Table S7).

**Supplementary Table S6. Basic demographic data of the validation sub-populations.** Sub-set 3 is part of sub-set 2, and sub-set 2 is part of sub-set 1. CHD: congenital heart defect

|  | Sub-set 1: Automatic volumetry validation dataset | Sub-set 2: Manual stereology of cerebellum, total brain volume and intracranial volume | Sub-set 3: Stereology incl. cortex and white matter volumes |
| --- | --- | --- | --- |
| Number of cases | 52 | 40 | 28 |
| Male/female | 34/18 | 28/12 | 17/11 |
| CHD / controls | 35/17 | 30/10 | 18/10 |
| Corrected gestational age at time of MRI (weeks, mean ± SD, range) | 41.4 ± 2.1,  37.6 – 46.2 | 41.2 ± 1.9,  37.6 – 45.1 | 41.3 ± 1.9,  38.1 – 45.1 |

***Image processing***

The validation was carried out on the reconstructed 3D T2 images. First, non-brain tissue parts of the axial, coronal and sagittal T2-weighted images were removed by applying image masks generated from an age-specific neonatal neuroanatomical atlas. The masked, axial and coronal images were co-registered to the sagittal image using a mutual information based affine and non-linear registration as implemented in the Niftireg image registration package (Modat et al., 2010). Bias field correction of the images was performed by the N4ITK filter in the Slicer 3D software (Fedorov et al., 2012), and the three orthogonal resolution images were resampled to form a joint, high-resolution image. Examples for the reconstructed images and segmentations are given in the main manuscript.

***Automatic segmentation by non-linear transformation of anatomical priors***

Anatomical priors from the ALBERTs^[[1]](#footnote-1)^ data set, which is gestational age-specific neonatal anatomical atlas (Gousias et al., 2012), were matched to the subjects reconstructed images using a linear affine registration (flirt in FSL) followed by a fast freeform non-linear deformation (reg_f3d command in the Niftireg software), based on the algorithm of Modal et al. 2010. The algorithm uses cubic B-spline to deform a source image in order to optimise an objective function based on the normalized mutual information and a penalty term. The penalty term used was the bending energy.

The parameters of the non-linear registration were the following:

- Grid spacing along the x,y,z directions: 8 * 8 * 8 mm
- Penalty term: bending energy=0.005

The anatomical masks of the supratentorial white matter, grey matter (cortex only) and lobar subdivisions of the supratentorial white matter (frontal, parietal, temporal and occipital lobe) were matched to the T2-weighted super-resolution images. A whole-brain mask was defined to include the diencephalon, cortical mantle, supratentorial white matter, but not the intra- and extra axial cerebrospinal fluid spaces or cerebellum. The patient-specific priors were thresholded at 50 % probability, and their volume was stored for the statistical evaluations.

***Validation using the automatic segmentation by dHCP pipeline***

The dHCP structural pipeline was used to automatically segment the neonatal brain (Makropoulos et al., 2018). This pipeline is a fully automated processing pipeline for T1 and T2 weighted neonate brain MRI images. The pipeline performs cortical and sub-cortical volume segmentation, cortical surface extraction, and cortical surface inflation. The segmentation is based on an adaptive Expectation-Maximization algorithm (Makropoulos et al., 2014, 2016).

***Manual volumetry***

Manual validation was performed by stereology using the EasyMeasure software package (Puddephat, 1999), following a method that has previously been described in detail elsewhere (Mayer et al., 2016). During stereology, a random grid is overlaid on the MR image, and the user assigns a label to each grid point that belongs to a given structure (Supplementary Figure S2).

***
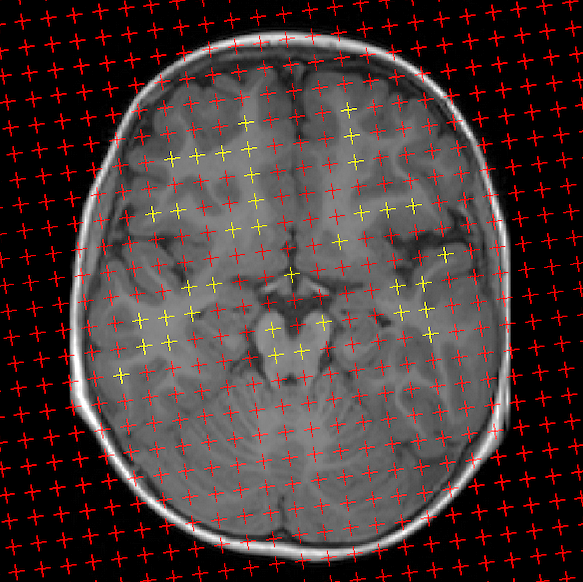
***

**Supplementary Figure S2. Manual volume estimation by stereology in the EasyMeasure software.** Grid points are displayed in red, overlaid on a T2-weighted neonatal MR image. (Illustration not actually used for measurement)

***Correlations and overlaps of regional volumes across methods***

The agreement of the two automated volumetry approaches with ground truth manual stereology data was tested by calculating the correlation coefficient (Supplementary Table S8).

**Supplementary Table S7. Estimation of regional brain volumes and correlations with manual, ground truth stereology.**

| Region, method | Correlation coefficient (Pearson’s r) | Significance (p) |
| --- | --- | --- |
| Total brain volume, Atlas^1^ | 0.930 | 4.56*10^-18^ |
| Cerebellum, Atlas^1^ | 0.827 | 4.89*10^-11^ |
| Cortex, Atlas^1^ | 0.881 | 6.27*10^-10^ |
| White matter, Atlas^1^ | 0.675 | 0.000111 |
| Cerebellum, dHCP^2^ | 0.914 | 4.53*10^-16^ |
| Cortex, dHCP^2^ | 0.954 | 1.18*10^-15^ |
| White matter, dHCP^2^ | 0.566 | 0.002 |

*1: Atlas prior based approach, used during the main hypothesis test.*

*2: Method by Makropoulos et al.*

As stereology does not result in binarized label maps, we were only able to calculate the spatial overlaps between the two automated approaches. Volumetric overlap was estimated as the Dice coefficient (%) of the subject-matched anatomical priors (binarized at 50%) and the transformed label maps of the dHCP pipeline. Table S2/3. summarizes the accuracy of the atlas prior based approach to the dHCP pipeline and manual stereology.

**Supplementary Table S8. Correlation of volume estimates and spatial overlaps with the atlas prior based approach.**

| Region, method | Correlation of volume estimates (Pearson’s r) | Correlation of volume estimates, significance (p) | Volumetric overlap (Dice coefficient, %) |
| --- | --- | --- | --- |
| Cerebellum, dHCP^1^ | 0.940 | 1.53*10^-24^ | 90.7 ± 2.2 |
| Cortex, dHCP^1^ | 0.877 | 3.44*10^-17^ | 74.1 ± 2.6 |
| White matter, dHCP^1^ | 0.745 | 3.67*10^-10^ | 80.4 ± 2.4 |
| Cerebellum, Manual | 0.856 | 3.55*10^-12^ | - |
| Cortex, Manual | 0.881 | 6.27*10^-10^ | - |
| White matter, Manual | 0.675 | 0.000111 | - |
| Frontal WM, dHCP^1^ | 0.511 | 0.000108 | 82.7 ± 3.2 |
| Parietal WM, dHCP^1^ | 0.547 | 0.000028 | 76.4 ± 6.3 |
| Temporal WM, dHCP^1^ | 0.887 | 4.54*10^-18^ | 72.7 ± 2.8 |
| Occipital WM, dHCP^1^ | 0.737 | 4.54*10^-10^ | 72.4 ± 3.4 |

*1: Method by Makropoulos et al.*

***CHD / control volumetric ratios***

We tested whether the CHD to control ratio depends on the utilized segmentation or stereology approach (Figure S3).


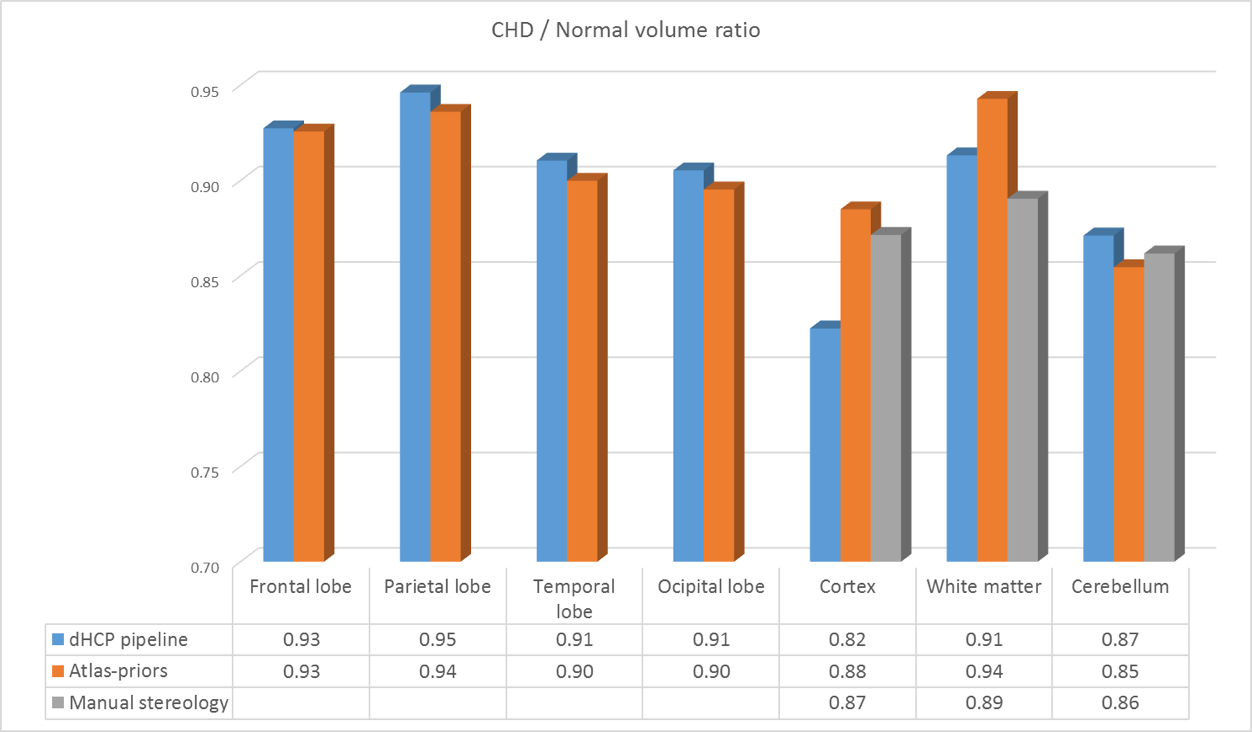


**Supplementary Figure S3. Comparison of the CHD-to-normal brain volume ratios across methods.** Lobar volumes refer to lobar white matter volumes.

***Validation of the atlas prior based approach - summary***

The anatomical atlas prior based segmentation and the dHCP pipeline gives comparable and faithful volumetry results for the cortex, total brain and cerebellar volume. The cortex and white matter volumes show larger variability, and less faithful estimation of the biologically relevant CHD/normal volume ratio. The volumes of lobar subdivisions show substantial differences between the methods (correlation coefficient 0.511-0.887), however, this could stem from the different anatomical definition of the deep white matter borders of the lobes in the two parcellation schemes. Despite the lower correlation of the lobar volumes, the corresponding CHD-to-normal ratios are remarkably similar between the two methods (difference: <2 %), while the cortex / white matter boundaries differed between these methods by a margin of 3-7%. The high variability of cortical volumes by both automated approaches limit the generalisability of the volumetric findings, and more reliable and reproducible results can be expected for lobar white matter, total brain volume and cerebellar volume estimates.

**References**

Fedorov A et al. 3D Slicer as an image computing platform for the Quantitative Imaging Network. Magn Reson Imaging. 2012;30(9):1323-41.

Gousias ISet al. Magnetic resonance imaging of the newborn brain: manual segmentation of labelled atlases in term-born and preterm infants. Neuroimage. 2012;62(3):1499-509.

Makropoulos, A. et al, D. (2014). Automatic whole brain MRI segmentation of the developing neonatal brain. IEEE Trans. Med. Imaging 33, 1818–1831.

Makropoulos, A. et al. (2016). Regional growth and atlasing of the developing human brain. NeuroImage 125, 456–478.

Makropoulos, A. et al. (2018). The developing human connectome project: A minimal processing pipeline for neonatal cortical surface reconstruction. NeuroImage 173, 88–112.

Mayer KN.et al. Comparison of automated brain volumetry methods with stereology in children aged 2 to 3 years. Neuroradiology. 2016;58(9):901-10.

Modat M. et al. Fast free-form deformation using graphics processing units. Comput Methods Programs Biomed. 2010;98(3):278-84.

Puddephat MJ. Computer interface for convenient application for stereological methods for unbiased estimation of volume and surface area : studies using MRI with particular reference to the human brain. [electronic resource, <https://ethos.bl.uk/OrderDetails.do;jsessionid=CF6826CCA0D06D076AAEB37BAFF44E53?uin=uk.bl.ethos.368022>, accessed: 23. 11. 2018]: University of Liverpool; 1999.

1. Copyright Imperial College of Science, Technology and Medicine and Ioannis S. Gousias 2013. All rights reserved. [↑](#footnote-ref-1)
